# Right-sided cardiogenic shock from acute pulmonary tumor thrombotic microangiopathy: a rare but deadly cardio-oncologic and metabolic emergency

**DOI:** 10.1007/s00392-025-02746-w

**Published:** 2025-09-15

**Authors:** Stefanie Andreß, Rima Melnic, Hannes Christow, Dominik Buckert, Philipp Marcel Jan Mohr, Benjamin Mayer, Wolfgang Rottbauer, Armin Imhof, Sascha d’Almeida

**Affiliations:** 1 Department of Internal Medicine II, University Heart Center Ulm, Ulm University Hospital, Ulm, Germany; 2 Department of Cardiology, Nephrology, Angiology, Pneumology and Intensive Care Medicine, Heidenheim Hospital, Heidenheim, Germany; 3 Department of Diagnostic and Interventional Radiology, Ulm University Hospital, Ulm, Germany; 4 Institute for Epidemiology and Medical Biometry, Ulm University, Ulm, Germany

**Keywords:** PTTM, Pulmonary Tumor Thrombotic Microangiopathy, PH, Pulmonary Hypertension, CUP, Cancer of Unknown Primary, Adenocarcinoma, Post-mortem, Sudden cardiac death, Right heart failure, Metabolic syndrome, SPAP, systolic Pulmonary Artery Pressure

## Abstract

**Background:**

Pulmonary tumor thrombotic microangiopathy (PTTM) is a fatal but treatable condition characterized by the rapid development of pulmonary hypertension (PH) in patients with possibly unknown adenocarcinoma. PTTM is mostly diagnosed post-mortem and considered a rare disease since its acute onset and misdiagnosis provides significant diagnostic and therapeutic challenges.

**Methods:**

We conducted a retrospective analysis of patients who presented with unclear sudden cardiac death and acute right heart failure that had an incidental very recent or unknown malignant cancer, identified eight patients with PTTM and reported the results. Patients were considered from 2009 to 2024 and analyzed at Ulm University Heart Center, Germany with the aim to describe the fatal consequences of unknown acute PTTM with right heart failure and discuss diagnostic and therapeutic strategies.

**Results:**

The median age was 47 years (41–84 years); gender was equally distributed. The latest median body mass index (BMI) was elevated with 28.4 kg/m^2^ (25–36 kg/m^2^). All patients presented as an emergency and died in our hospital due to right heart failure caused by adenocarcinoma in various locations. Median high-sensitivity troponin T was elevated (42.5 (3–179, normal < 14) ng/L), median NT-pro-BNP (5375 (3100–14,000), normal < 800 for all age groups, in pg/mL), and d-dimer values (7.74 (1.1–21), normal < 0.5 for patients younger than 50 years and < 1 for all other age groups, in mg/FEU) were strongly elevated. Median HbA1c was slightly elevated 7.4% (normal < 6.5%). Median time from last hospital admission to death was 8 days (1–23 days). At admission, median systolic arterial pressure (sPAP) estimated by echocardiography was 65 (46–115) mmHg. Low NT-proBNP and sPAP values as well as pre-mortem adenocarcinoma diagnosis and (therewith associated) adenocarcinoma-type cancer of unknown primary (CUP) correlated best with longer survival in days (*ρ* and *r*-values: − 0.88, − 0.76, 0.58, 0.89 respectively). Initiation of specific therapy (chemotherapy or anticoagulation) was correlated with survival (*ρ* = 0.786, *p* = 0.02).

**Conclusion:**

Our data suggest that the combination of elevated hsTnT, NT-proBNP, d-dimer, and HbA1c values in patients with unexplained acute right heart failure may indicate PTTM. Our findings also emphasize the diagnostic challenge posed by PTTM, and imply that targeted therapy, enabled by a timely diagnosis, may improve survival. Therefore, acute and fatal right heart failure in the adult in absence of coronary artery disease, pulmonary embolism, or any other apparent cause, especially in patients with uncontrolled metabolic syndrome, should prompt an urgent diagnostic work-up to rule out unknown cancer with treatable pulmonary tumor embolism, beginning with more extensive imaging (e.g., computed tomography (CT) and magnetic resonance tomography (MRI)), as well as laboratory diagnostics (e.g., tumor markers). In still inconclusive cases, lung biopsy and right heart catheterization should be considered eventually, if possible.

**Graphic Abstract:**

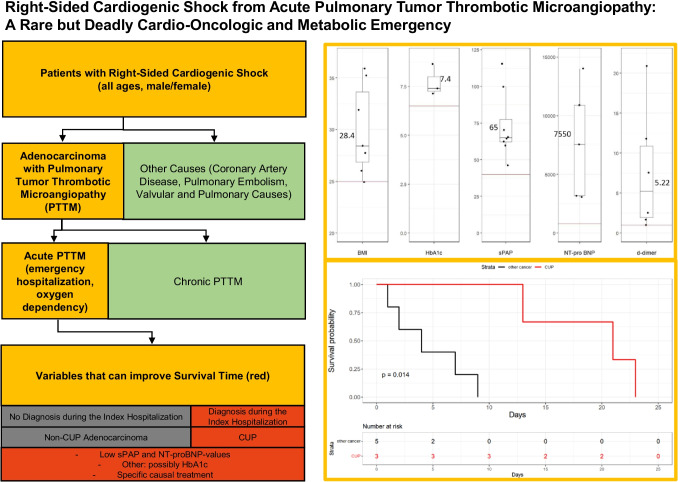

## Background

### Description

Pulmonary tumor thrombotic microangiopathy (PTTM) is considered a very rare complication of untreated or terminal adenocarcinoma. Mainly because of its anatomical proximity and incidence, gastric adenocarcinoma has been described as the leading malignant entity leading to PTTM [1, 2]. Despite possible bias in data collection, the largest metanalysis published yet estimated that it causes around 60% of PTTM cases [1]. In addition, PTTM mainly occurred in breast cancer, lung cancer, and urothelial cancer but case reports include almost every type of adenocarcinoma [1–5]. In literature, about 110 case reports with around 200 patients (mostly with three or less patients per report) have been published (as of 2024), and most cases are diagnosed post-mortem as the mean time from symptom onset to death has been estimated to be about 9.5 weeks [1]. Especially in patients presenting with acute PTTM, prognosis is poorer.

### Pathophysiology and symptoms

Through a yet unknown mechanism, tumor cells spread into the pulmonary circulation and cause an obstruction disproportionately affecting small pulmonary arteries [1, 6], which translates into symptoms such as hemoptysis, cough, dyspnea, and sometimes chest pain that can be easily mistaken for other internal diseases. This process leads to microthrombi, endothelial damage, and remodeling [7]. Pulmonary infiltration can be measured by a sudden rise in systolic arterial pressure (sPAP) but remains concealed in conventional computed tomography (CT). The differential diagnosis of pulmonary hypertension (PH) includes complex and time-consuming diagnostic procedures [3] such as transthoracic echocardiography (TTE) and right heart catheterization (RHC) and their correct interpretation, which explains why a rapid progressive (acute) PTTM shows a high lethality.

Because of the acute onset of symptoms most cases are diagnosed post-mortem. Therefore, efforts must concentrate on reducing the time to diagnosis. A systematic review that analyzed 160 reported cases from 79 studies to identify common clinical features, reported imaging findings, and laboratory abnormalities associated with PTTM [1]. Dyspnea (94%) and cough (85%) were the most frequent presenting symptoms, while hypoxemia was present in 95% of cases. Elevated D-dimer (95%), anemia (84%), and thrombocytopenia (77%) were common laboratory findings. Imaging typically revealed ground glass opacities (82%) and pulmonary nodules (86%). In TTE, PH was noted in 89% of cases, with an average sPAP estimation of 71 mmHg [1].

Given the latest breakthroughs in late cancer research resulting in patients living longer with cancer, the incidence of PTTM is expected to rise [8], and its acuity is not thoroughly reflected in the current German Guidelines for PH [9].

### Diagnosis

**Fig. 1:**
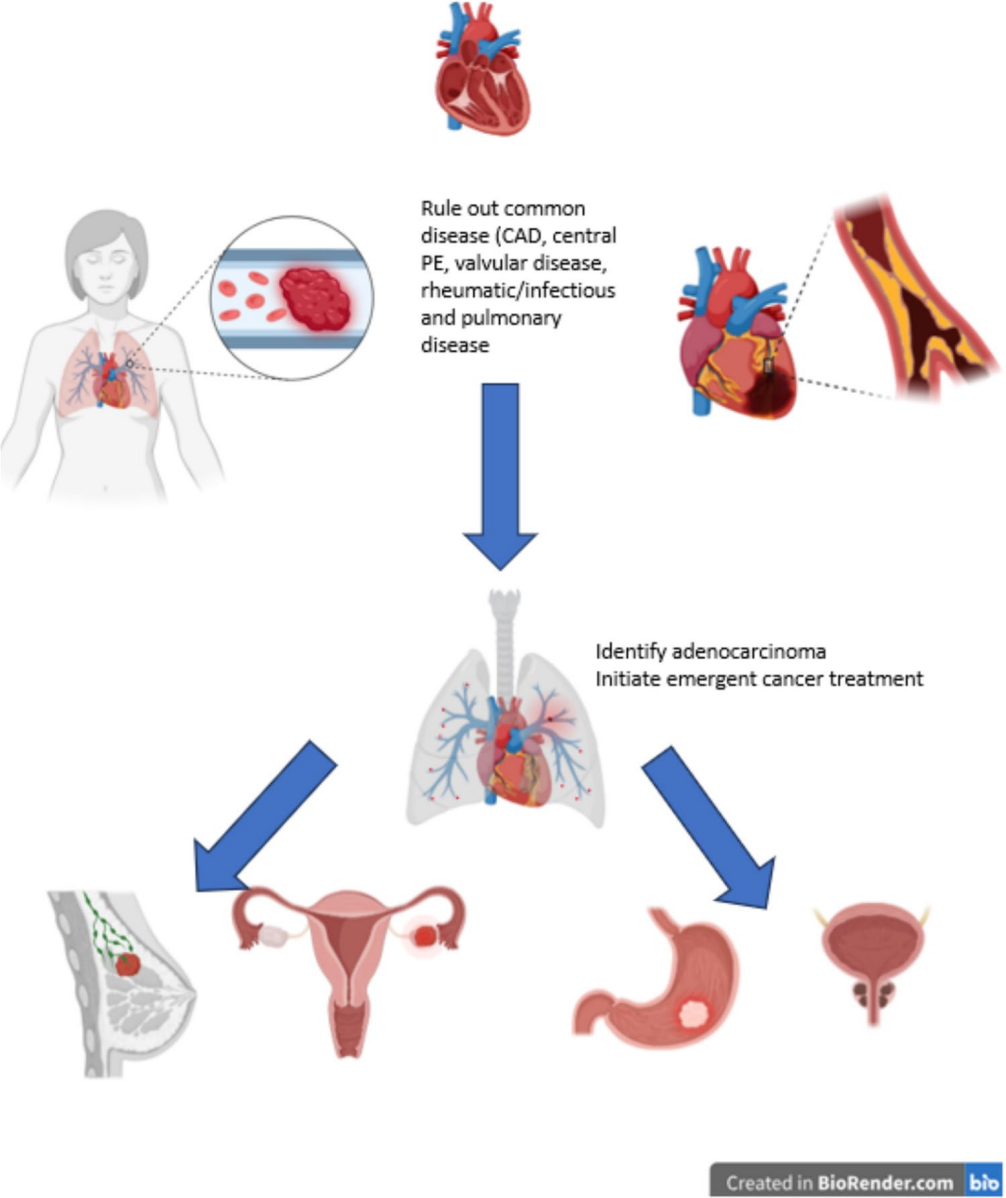
Diagnostic algorithm for detection of PTTM in acute right heart failure: patients with acute right heart failure were screened for having PTTM. Patients eligible for inclusion underwent a step-by-step diagnostic algorithm with exclusion of other reasons for pulmonary hypertension such as CAD, central PE, valvular disease, rheumatic/infectious and pulmonary disease, which was followed by a search for cancer. Finally, acute right heart failure patients without other causes and with cancer diagnosis were diagnosed with PTTM

A possible diagnostic approach for PTTM is shown in Fig. 1 and demands the collaboration of cardiology, pulmonology, radiology, oncology, and pathology. Patients with acute right heart failure measured mostly by sPAP and that do not have a coronary artery disease (CAD), a valvular disease, a central pulmonary embolism (PE), a rheumatic or infectious disease should be screened for unknown cancer.

Regarding PTTM itself, lung biopsy or dual-energy CT is possible tools to diagnose PTTM in the living. In the acute setting, lung biopsy is complicated by the right heart failure and by the time needed by the pathologist to process the histology. Normal CT protocols mostly miss the primary tumor but detect ground glass opacities, nodules, and mediastinal lymph nodes [1] which are unspecific changes. In addition, possible peripheral pulmonary emboli caused by tumor growth might be misleading. Wedge shaped defects are a useful radiologic finding [3] but require a dual-energy protocol which is not necessary for detecting PE and is not routinely available in most emergency situations.

Lab parameters are known to include slightly elevated BNP values, elevated d-dimer values, and changes in blood smear sometimes with signs of disseminated intravascular coagulation (DIC) [1, 5].

## Methods

### Study design

In this study, we retrospectively searched for patients that presented with unclear sudden cardiac death and acute right heart failure and had an incidental very recent or unknown malignant cancer and reported the results. Patients were considered from 2009 to 2024 and analyzed in the University Heart Center in Ulm Germany. Within all cases, eight were identified of clearly having acute PTTM as cause of death and we assessed all clinical findings, especially the past medical history, lab values, radiological findings, and the results of post-mortem sections, when available. Acute PTTM was defined by oxygen dependency at admission, which is associated with a particularly high mortality [1]. All patients were diagnosed with adenocarcinoma in various locations.

Patients with known and stable PH were ruled out when they did not have a post-mortem section. Patients with central PE were ruled out. If PTTM was detected in a post-mortem autopsy, patients with segmental or subsegmental PE were included as we considered the subsegmental embolism to be a thrombus formation due to PTTM.

### Clinical assessment and data collection

We assessed all clinical findings, in particular medical history, laboratory values, radiological findings and the results of autopsies, when available. Additionally, we assessed specific therapies including antineoplastic therapy such as chemotherapeutics and radiation, symptomatic therapy for pulmonary hypertension and anticoagulation along with their starting point, and separated into newly initiated during the index hospitalization or pre-existing. We then calculated the effect of new initiation of specific therapy on survival. Information was extracted from our clinical information system (SAP).

Left ventricular ejection fraction (LVEF) was measured by echocardiography (EPIQ 7, Koninklijke, Philipps N.V. Eindhoven, Netherlands) and categorized in hypercontractile (> 75%), normal (55–74%), mildly reduced (35–54%), and reduced (< 35%). High-sensitivity troponin T (hsTNT), NT-pro BNP, and d-dimer levels were measured from blood samples (ElectroChemiLuminiszenzImmunoAssay, ECLIA Roche Cobas 8000, Modul E801 and E601).

### Endpoints

The primary endpoint were abnormal baseline characteristics, which were reported descriptively. Secondary, we assessed predictors of survival time. Baseline characteristics, time of adenocarcinoma diagnosis, and adenocarcinoma type were tested as potential predictors. The observation period lasted from admission to our hospital until death.

### Statistical analysis

Because of the small number of patients, continuous variables were presented as median values together with the overall range of the collective. Categorial variables were described as numbers and percentages. The Mann–Whitney *U* test was used to compare variables between groups where appropriate. Spearman’s correlation (*ρ*) was used to analyze non-categorial parameters that could correlate with survival length after presentation. Point biserial correlation (*r*) was used to analyze the correlation of non-categorial variables with survival length. Kaplan–Meier estimator was used to assess the time to death and comparison of groups was performed using the log rank test. The proportional hazard assumption was tested using Schoenfeld residuals. Values for body mass index (BMI) were calculated with the latest weight available, which includes B-symptom-triggered weight loss.

This study was approved by the local Ethics committee of the University in Ulm and adheres to the Declaration of Helsinki. All data was collected as part of clinical routine. We did not perform any additional exam in order to collect the data. Due to the explorative nature of this study, all results of statistical tests have to be interpreted as generating hypotheses.

## Results

### Baseline characteristics

With the methods described above we identified eight patients with PTTM. Baseline parameters of all patients are displayed in Table 1. Median age was 47 years (41–84 years); sex was equally distributed; and the median latest BMI was 28.41 kg/m^2^ (25–36 kg/m^2^). All patients presented as an emergency and died within short periods in our hospital. Median time from hospitalization to death was 8 days (1–23 days). Median sPAP at admission was 65 (46–115) mmHg. LVEF was hypercontractile in two cases, normal in three cases, mildly reduced in two cases and severely reduced in one case (history of known dilatative cardiomyopathy). PE was ruled out in six cases, and full-dose oral anticoagulation had been prescribed for at least 3 months in the other two cases.

**Table 1:** Itemized breakdown of baseline characteristics (assessed at admission)

Patient	1	2	3	4	5	6	7	8	Med	ρ/r*
Age	63	41	46	46	47	72	84	46	47	0.37
Gender	Male	Female	Male	Female	Female	Female	Male	Male		0.62*
Body height*	169	165	183	165	165	167	174	x	167	
Weight*	101	68	86.5	98	76	89	86	x	86.5	
BMI	35.36	25.00	25.00	36.00	27.92	31.91	28.41	x	28.41	
Days from admission to death	13	7	1	9	23	21	4	3	8	
sPAP	70	63	115	65	46	60	65	100	65	− 0.76
LVEF	HFrEF	HC	Normal	HFmrEF	Normal	HC	HFmrEF	Normal		0.17
PE exclusion*	Yes	Yes	Yes	Yes	Yes	No	No	Yes		
Diagnosis of Adeno Carcinoma	Pre-mortem	Pre-mortem	Post-mortem	Post-mortem	Pre-mortem	Pre-mortem	Pre-mortem	Post-mortem		0.58*
Localization	CUP/renal	Mammal	Gastric	Ovarial	CUP	CUP	Prostate/colon	Bladder		0.89*

Height measured in cm, weight measured in kilogram. *BMI*, body mass index; sPAP, systolic pulmonary arterial pressure; *LVEF*, left ventricular ejection fraction; *PE*, pulmonary embolism; *CUP*, cancer of unknown primary; *HFrEF*, heart failure with reduced ejection fraction; *HFmrEF*, heart failure with mildly reduced ejection fraction; *Med*, median; *ρ*, Spearman’s correlation coefficient, all patients without PE exclusion were on sufficient oral anticoagulants. Patient 7 had acetylsalicylic acid (ASS) additionally. *ρ* = Spearman correlation of parameters with survival time * point biserial correlation of parameters with survival time**,** in patients 2 and 7 adenocarcinoma was known before index presentation

### Lab values

**Fig. 2:**
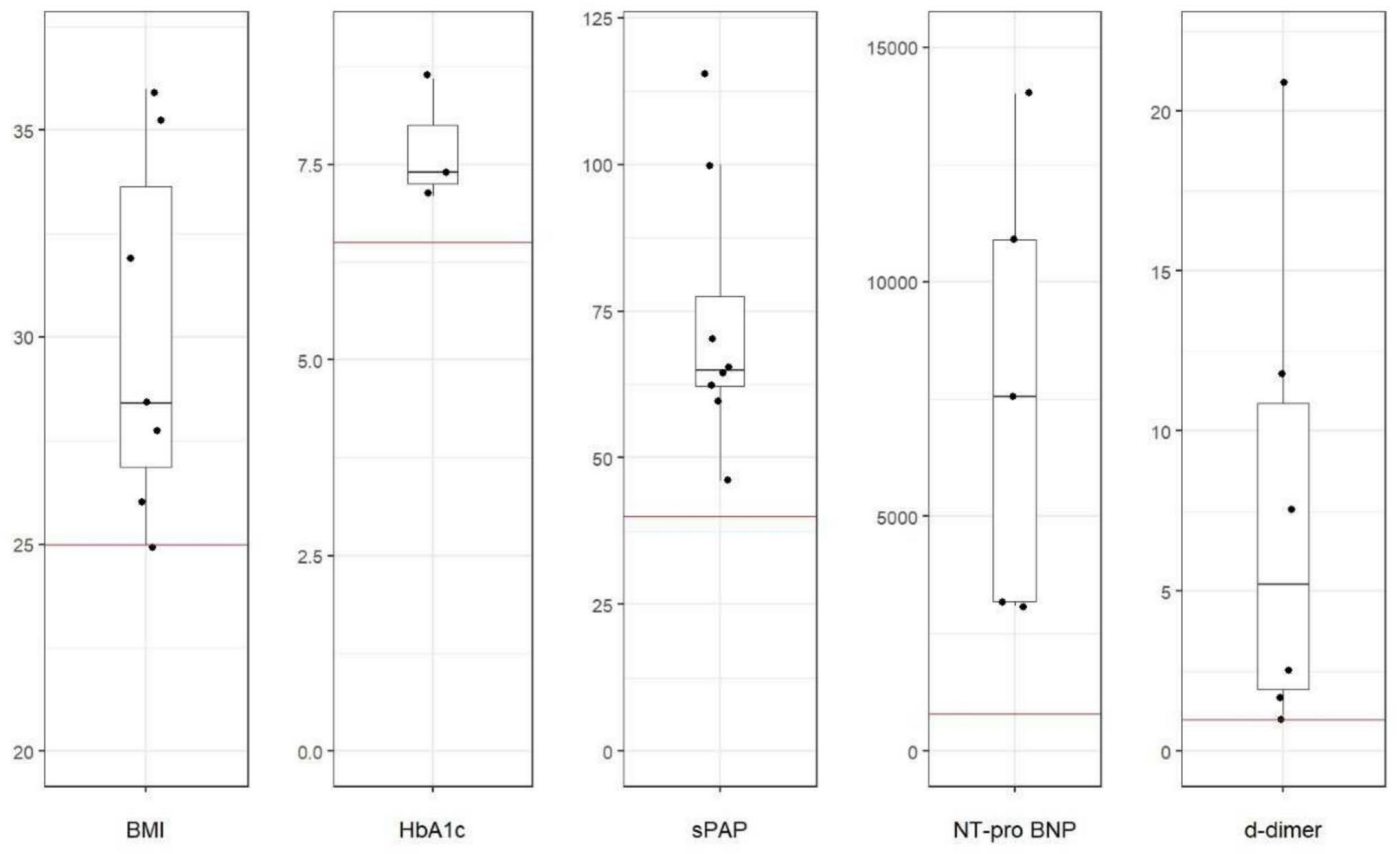
Important parameters in PTTM presented in a box blot. Abnormal clinical characteristics in patients with PTTM at admission relative to standard cutoff values. Median BMI, HbA1c, sPAP, NT-proBNP, and d-dimer values were elevated and all of these parameters were consistently elevated across all patients. *BMI is expressed in kg/m^2^, Hba1c in %, sPAP in mmHg, NT-proBNP in pg/mL, d-dimer in mg/FEU). The red line value represents the normal cutoff value, the box represents the median value and interquartile range for PTTM patients. It is noteworthy that all PPTM patients were above the cutoff value for each of the parameters stated; PTTM, Pulmonary tumor thrombotic microangiopathy; sPAP, systolic pulmonary artery pressure

An itemized breakdown of lab values is displayed in Table 2. Median hsTnT was elevated with 29 (3–179, normal < 14) ng/L, median NT-pro-BNP was 7550 (3100–14,000), normal < 800 for all ages) pg/mL and d-dimer values were 5.22 (1.1–21) (normal < 0.5 for under 50 years old patient and < 1 for all ages) mg/FEU, and median HbA1c was 7.4% (normal < 6.4). For all patients, NT-proBNP and d-dimer values were elevated. Abnormal values of baseline parameters in our population are displayed in Fig. 2.

**Table 2:** Itemized breakdown of all patients’ lab values (assessed at admission)

Patient	1	2	3	4	5	6	7	8	Med	ρ/r*
hsTNT*	3	179	56	29	n.m.*	107	19	12	29	0,072
NT-proBNP	n.m	7550	n.m	3100	n.m	3200	14000	10895	7550	− 0.87
D-Dimer	11.9	21	1.1	2.69	n.m	n.m	7.74	1.67	5.22	0.44
Creatinine	197	67	121	75	146	176	104	135	128	0.47
AST*	31	353	33	27	45	112	40	x	40	− 0.14
ALT*	14	125	n.m	49	34	20	52	33	34	− 0.39
GGT*	241	313	77	183	459	328	n.m	69	241	0.87
LDH*	153	2500	235	203	185	1652	430	1797	332.5	− 0.35
HBA1c*	7.4	n.m	n.m	n.m	n.m	7.1	8.6	x	7.4	− 0.88
Glucose*	214	118	61	95	82	222	303	147	132.5	− 0.12
WBC*	7.5	12.6	14.8	10.1	21.7	7.8	12.8	14.6	12.7	− 0.34
PLT*	345	80	274	256	323	168	254	155	255	0.23
Hb*	8.8	13.9	13.5	13.4	10	10.4	13.1	12.2	12.65	− 0.66
CRP*	285.9	24.8	27.1	34.6	246.7	55.7	98.2	290.0	76.95	0.34

*All values at admission, except for HbA1c if less than four weeks old. n.m. = not measured, hsTnT, high-sensitivity troponin T; *AST*, aspartate transaminase; *ALT*, alanine transaminase, gamma glutamyl transaminase; *LDH*, lactate dehydrogenase (< 250 U/L); *HbA1c*, glycated hemoglobin fraction (< 6,4%); *WBC*, white blood cell count (< 10.5 G/L); *PLT*, platelet count (150–350 G/L); *Hb*, hemoglobin (12–17 g/L); *CRP*, C-reactive protein (< 5 mg/L); glucose = earliest glucose value measured (60–125 mg/dL); *Med*, median; *ρ*, Spearman’s correlation coefficient

### Course of the disease

Diagnosis of adenocarcinoma was made in two patients shortly before admission, during the clinical stay in three patients and in three patients post-mortem. Cancer was without known primary in three times (CUP), kidney cancer, breast cancer occurred once, and gastric, ovarial, colon, prostate, and bladder adenocarcinoma (multiple localizations per patient possible). Patients with CUP syndrome had a less aggressive form of PTTM. In three patients, mean survival was 21 (13–23) days, while for non-CUP patients it was four (1–9) days (*p* = 0.036). Patients that were diagnosed pre-mortem had a median survival of 13 days, patients with post-mortem diagnosis had a median survival of three days (*p* = 0.143).

### Predictors of survival length

The parameters that correlated with the length of survival after admission were the achievement of the cancer diagnosis during the index hospitalization which corresponded to a diagnosis of adeno-CUP (*r* = 0.892, log rank *p* = 0.014, the level of gamma GT (*ρ* = 0.87), NT-proBNP levels (*ρ* =  − 0.87), and the estimation of sPAP values (*ρ* =  − 0.76) in TTE. Pre-mortem diagnosis of adenocarcinoma correlated with a longer survival time (*r* = 0.58), especially when first diagnosed during the index hospital stay (as were all CUP syndromes, *r* = 0.892). Female gender correlated with a longer survival (*r* = 0.62), but two out of three CUP diagnosis were women. Creatinine levels (*ρ* = 0.47), age (*ρ* = 0.37), and d-dimer levels correlated only weakly (*ρ* = 0.44) with survival even though all isolated d-dimer values were higher than the cutoff. Markers of left ventricular function were not conclusive. HsTNT (*ρ* = 0.07) and LVEF (*ρ* = 0.17) did not correlate with survival length.

### Treatment of PTTM

To further evaluate the currently available therapeutic options in clinical practice, we collected data on patients’ specific therapy for PTTM. Notably, in all three patients who were diagnosed with cancer during the index hospitalization, a specific therapy was started. Patient 5 received chemotherapy with etoposide and cisplatin; in patients 1 and 6, therapeutic anticoagulation was initiated. Patient 6 was additionally recommended to start chemotherapy and radiotherapy, but declined these therapies. In the two patients who presented with known cancer, there was no change in therapy during the index hospitalization. In patient 2, rescue-chemotherapy was planned, but the patient died before this could be done. Patient 7 was already on anticoagulation due to atrial fibrillation. In the three patients who were diagnosed with cancer post-mortem, one was started with anticoagulation due to the suspicion of WHO class I PH, in the other two specific therapy remained unchanged. Patient 3 was already under anticoagulation due to a history of thrombosis. Notably, all and only patients in whom specific therapy was newly initiated, survived longer than one week after admission. Accordingly, initiation of specific therapy was correlated with survival (*ρ* = 0.786, *p* = 0.02). The longest survival was observed in patient 5, who was started with combined cancer treatment with chemotherapy and radiation. Patients’ therapies in detail along with the timepoint of initiation are shown in Table 3.

**Table 3:** Itemized breakdown of specific therapy of PTTM along with the starting point

Patient	1	2	3	4	5	6	7	8
Chemotherapy	No	No (planned)	No	No	Yes (Cisplatin, Etoposide)	No (refused)	No	No
Start during index Hospitalization	No	No	No	No	Yes	No (refused)	No	No
Tyrosine kinase inhibitor	No	No	No	No	No	No	No	No
VEGF inhibitor	No	No	No	No	No	No	No	No
Radiation	No	No (planned)	No	No	Yes	No (refused)	No	No
Start during index Hospitalization	No	No	No	No	Yes	No (refused)	No	No
Anticoagulation	Yes	No	Yes	Yes	No	Yes	No	No
Start during index hospitalization	Yes	No	No	Yes	No	Yes	No	No
Vasodilators	No	No	No	No	No	Yes (Bosentan, Sildenafil)	No	No
Start during index Hospitalization	No	No	No	No	No	No	No	Yes
Start of specific therapy (chemotherapy or anticoagulation)	**No**	**No**	**No**	**Yes**	**Yes**	**Yes**	**No**	**No**
Correlation with survival time (days)	**Spearman’s correlation coefficient *ρ* = 0.786, *p* = 0.02**							

## Discussion

### Summary

This study sought to identify and describe characteristics of PTTM with a focus on identifying abnormal clinical characteristics of patients with PTTM and predictors of survival length. We retrospectively identified eight patients with PTTM and demonstrated that it is a deadly complication of adenocarcinoma of various locations and presents with acute right heart failure in patients of every age. All patients described here died within 23 days. In our eight-patient population, BMI, sPAP, d-dimer, NT-proBNP, and CRP were elevated. Low NT-proBNP and sPAP correlated best with survival. Indicators usually used for CAD diagnostics like hsTNT of LVEF were not conclusive as to the length of survival. Low platelets as a manifestation of DIC were only apparent in one of eight patients. Pre-mortem diagnosis of cancer (including diagnosis before the index hospitalization) showed a positive correlation with survival (*r* = 0.58), but the sample size was not large enough to prove this correlation with Mann–Whitney-*U* test (*p* = 0.143). Pre-mortem diagnosis within the index hospitalization, which was exclusive to all patients with CUP, correlated better with survival (Spearman *ρ* 0.89, log rank 0.014). It is arguable that patients with CUP have a less aggressive form of PTTM or CUP is diagnosed more easily. Initiation of specific therapy was correlated with survival (*ρ* = 0.786, *p* = 0.02). Still, because of the emergent referral and oxygen dependency at admission it was an advanced stage of acute PTTM with a poor prognosis in all patients. We also observed a correlation between female sex and longer survival. This may be related to a confounding effect of CUP syndrome mostly being present and diagnosed pre-mortem in our female patients. Our data hints that achieving the diagnosis of adenocarcinoma in PTTM is associated with longer survival, which is more frequently achieved in CUP syndrome, and indicate that affected patients can be identified clinically by signs of right heart failure and metabolic dysregulation.

### The clinical view: PTTM is underdiagnosed

The short survival after diagnosis and the high proportion of patients who are not diagnosed with adenocarcinoma during their lifetime demonstrates the diagnostic challenge and consequently the underdiagnosis of adenocarcinoma complicated by PTTM. PTTM is challenging and its suspicion should induce a rapid diagnosis and treatment of cancer. If cancer is not apparent (especially in CUP syndrome) in a conventional scan or with lab values, diagnostic possibilities are very limited and include time-consuming procedures such as dual-energy CT-scan protocols, lung biopsy, and nuclear imaging. While in our study adeno-CUP was the most frequent type of adenocarcinoma, the largest review to date yet described gastric adenocarcinoma as main cancerous entity to develop PTTM. The authors admit that there is much possible bias in that observation [1, 7] especially due to possible underreporting of certain cancer types. Indeed, our study does only show one case of gastric adenocarcinoma. This may indicate that we are underreporting PTTM, and the incidence is higher than described here. PTTM has been described in up to 3% of autopsies of adenocarcinoma patients in general and in one of six patients (17%) with gastric adenocarcinoma in particular [1, 7]. By extrapolation, nine PTTM complications due to gastric adenocarcinoma are likely not to have been diagnosed. However, it is unclear if the undiagnosed cancer patients mostly died of or with PTTM. Only obduction series that clinically correlate right heart failure or right ventricular wall thickness with the occurrence of PTTM can differentiate between correlation and comorbidity. Predominance of gastric cancer in PTTM findings can also be caused by the anatomical proximity (for metastasis) to the lung. Patients with gastric cancer experience excessive weight loss, both due to tumor progression and loss of appetite, possibly leading to slower tumor growth and a longer timeframe for the development of PTTM. In contradiction to CUP syndrome, gastric cancer can more often be an incidental finding of a chest CT-scan, performed because of dyspnea and cough.

In our small population, it remains unclear if CUP syndrome-caused PTTM has a slower progression, or the patients develop the symptoms earlier. This highlights the heterogeneity of PTTM. Still, the data suggests that reaching the diagnosis as it was possible with CUP syndrome improves the survival time of patients. This makes PTTM and its diagnosis an emergency. A common point in the patients we observed is that acute PTTM patients tend to be adipose and to have a diabetes. The median BMI of 28 especially after B-symptoms caused weight loss as well as the median HbA1c of 7.4% indicate that acute PTTM might be linked to an uncontrolled metabolic syndrome. This correlation between cancer and obesity is also known as the so-called obesity paradox in cancer [10] and states that obese patients do have a higher risk of developing cancer, but B-symptoms and cancer-associated appetite loss normalize their weight before initial diagnosis [10]. The authors suggest a different assessment of body weight to prove the correlation to cancer [10]. Another approach could be a measurement of weight loss or weight delta per time unit.

In the following, we would like to further discuss that observation.

#### The rationale: The Warburg effect

The Warburg effect has been described almost 100 years ago [11, 12] and describes a particular anomalous metabolism of certain cancer cells. They tend to use more glucose than fatty acids to boost their metabolism and can therefore grow faster than other tissues. We have shown that patients with acute PTTM tend to have an uncontrolled metabolic syndrome, possibly associated with type 2 diabetes, and argue that long-time increased sugar levels in these patients lead to an easier metastasis and fuel the development of PTTM. The observation that diabetes increases the risk of cancer has already been shown in various studies and pointed out in consensus papers [12–14]. It is also known that insulin increases the risk, at least for certain cancer types [15]. In addition, the incidence of cancer in patients who use biguanides is lowered as demonstrated for liver and colorectal cancer [15], which might also be attributable to the competitive glucose metabolism in normal cells due to increased metabolism and therefore an indirect reduction of the Warburg effect.

We argue that non-adipose patients with PTTM might have a slower progression and have more time for their adenocarcinoma to be diagnosed and treated, so that acute right heart failure mainly puts patients with a fast progression and no cardiac reserve at risk. 

If so, another therapeutic strategy in PTTM might be the use of oral antidiabetics to reduce the glucose excess and slow down tumor metabolism. Because of the low rate of diagnosis, a study including PTTM patients will not be available soon. 

With only three HbA1c values obtained, the quality of Spearman *ρ* of − 0.89 is biased but a correlation can be suspected.

#### Lab results and differential diagnosis

Lab results can be misleading in PTTM. The more common diagnosis in known carcinoma associated with right heart failure is PE. A differential diagnosis in patients without adenocarcinoma is chronic thromboembolic pulmonary hypertension (CTEPH). Both entities show higher d-dimer values. Therefore, despite its high sensitivity, d-dimer is a poor predictor of PTTM due to its lack of specificity and its mediocre correlation to the severity of the disease. VEGF as a blood test was shown to have a good correlation [8] with the disease but was not tested in our acute setting. NT-proBNP shows a better correlation, especially in patients with normal LVEF. HsTNT does not correlate with the severity of the disease but still is a useful tool to rule out CAD, which is more commonly diagnosed. In the acute form of PTTM, thrombocytopenia is not common. In our population, one of eight patients had thrombocytes below 150 G/L, which is described in up to 77% of patients [1]. As of now, we cannot explain that high gamma GT values correlate best with the survival length, but we believe this finding to be accidental. Otherwise, a possible explanation is an early liver infiltration caused by the tumor leading to a faster referral to the emergency department. Here sPAP values can be used to assess the patient’s risk. These data show, that laboratory abnormalities caused by PTTM are unspecific, but the combination of the parameters indicating thromboembolic events and heart failure and the exclusion of the differential diagnoses may lead to a suspicion and diagnosis.

While individually nonspecific, the combination of elevated myocardial biomarkers, d-dimers, and HbA1c may represent a diagnostic signature for PTTM in patients presenting with acute right heart failure. Elevated hsTnT and NT-proBNP as well-established markers of myocardial strain and right ventricular dysfunction are commonly seen in PH [16, 17]. A recent study of patients with acute decompensated PH and CTEPH who required intensive care unit admission showed that 57.4% of the patients had increased hsTnT levels and that BNP levels were slightly elevated at 406 (198–623) pg/ml [18]. In our PTTM patient population, the increase of these myocardial biomarkers was more frequent and stronger, with 71.4% having elevated hsTnT and NT-proBNP levels of 7550 (3100–14,000), which underscores the severity of right heart failure in PTTM. D-dimers as a fibrin degradation product are an unspecific marker of macrothrombotic disease and therefore also predictive of venous thromboembolism such as PE [19], a common complication in cancer patients. Thus, they are less useful for differentiating the causes of PH; but, in the absence of PE, they may point towards tumor-associated microthrombosis within the pulmonary vasculature. However, metabolic dysregulation as evidenced by the consistently elevated HbA1c-levels observed in our PTTM patients, even in absence of known diabetes mellitus, is not associated with the risk of venous thromboembolism such as PE [20–22]. Since elevated HbA1c-levels are associated with chronic systemic inflammation, endothelial dysfunction, and procoagulant states [23, 24], they may indicate the presence of the paraneoplastic complication of PTTM. Taken together, the combined elevation of hsTnT, NT-proBNP, d-dimers, and HbA1c in patients with unexplained acute right heart failure may serve as a clinical clue for PTTM, and thus should prompt a search for malignancy. Given the high frequency of CUP syndromes in our study, it is open to discussion whether measurement of tumor markers could constitute a valuable additional diagnostic tool. The discovery of this unique biochemical pattern, which can be easily assessed based on blood samples, demonstrates the feasibility of diagnosing PTTM, even in the broad clinical setting of an emergency department.

The biochemical characteristics of patients with PTTM already suggest that this form of PH is particularly severe due to its cancer-associated pathogenesis. This is further supported by the finding that sPAP, which correlates with survival in CTEPH [25], has been reported to be even higher in CTEPH. Recent studies have reported sPAP values between 70 and 90 mmHg for these patients [26, 27], which is higher than the median of 65 mmHg in our population of PTTM patients. While survival is very short overall in PTTM, with the majority of patients dying before receiving specific therapy, and often even before the diagnosis, the prognosis in CTEPH patients has been significantly improved in the recent decades. An analogous Japanese study showed that in CTEPH the proportion of patients receiving specific treatment increased over the last decades to currently 97% [28] and, similarly, the 5-year survival rate rose from 68% at the end of the last century to 93% [28]. Even in a large study of patients with acute decompensation of pulmonary arterial hypertension and CTEPH who required intensive care unit admission, the 28-day survival rate was 60.3% [18]. These data impressively show that PTTM represents a unique pathology and consequently, despite similar clinical presentation, is fundamentally different to this other type of PH. Furthermore, PTTM’s particular dismissal prognosis is emphasized, which may be at least partly due to its diagnostic challenge.

#### Clinical course

Not only does sPAP correlate with the length of survival, the rise in sPAP can be a useful parameter to assess the progression of the disease [1]. This is confirmed in our population. Patient 7’s sPAP rose by 25 points after two days. In addition, sPAP is also a good long-term parameter. Indeed, in one case, sPAP had been monitored regularly because of a known and controlled class I PH caused by systemic autoimmune collagenosis. SPAP had been stable for almost eight years under PH triple therapy with bosentan, sildenafil, and Iioprost, and was monitored regularly. Unbeknownst to having cancer, the first relevant increase in sPAP was observed eight months prior to the lethal PTTM manifestation. With this case, we observed that PTTM caused lung obstruction can have a course of several months. The benefit of the medication remains unclear, as only singular case reports described an increase in survival with WHO class I PH therapy or other medication in PTTM.

#### Vasoactive and antiproliferative therapy

Various therapies have been tried in patients that were diagnosed pre-mortem including imatinib, tadalafil, bevacizumab, bosentan, oxaliplatin, epoprostanol, and oral anticoagulation but no regiment has been established yet [1, 29, 30]. The longest reported survival of 15 months was reached with combined chemotherapy (cisplatin/gemcitabine) and reported a regression of PTTM, followed by a lethal relapse [31]. Interestingly, a case with combined PDFG-inhibitor imatinib and VEGF inhibitor bevacizumab showed a similar result also culminating in a survival of twelve months followed by a relapse [29]. This indicates that tumor-adapted chemotherapy protocols may be a possible approach to tackle PTTM. A new approach from Chinese scientists with the new tyrosine kinase inhibitor anlotinib showed promising results as a potent way to reduce PH [32]. These data indicate that PTTM can be effectively treated and thus underline our findings that timely diagnosis is crucial to improve survival. An underestimated method for that purpose is the pulmonary wedge aspiration, an over 30-year-old RHC aspiration technique that can expose cancer cells in pathology. It has shown promising results in case reports [29, 33] and should be made available if unclear PH is diagnosed in RHC.

#### Clinical implications of diagnosing PTTM

Given the opportunity to start an adapted, specific therapy in PTTM, enabled by the feasibility of achieving a diagnosis during lifetime, we investigated the use and efficacy of the current therapeutic options. Fortunately, specific therapies already seem to be applied in clinical practice. In our population, all patients, who received the diagnosis of cancer in addition to PH, were started with specific therapy, including antineoplastic or antithrombotic therapy. One patient received chemotherapy, one refused it, and one was scheduled for chemotherapy. The fact that this patient died before starting chemotherapy suggests that the low proportion receiving chemotherapy may be due to the short survival time and poor clinical condition of our PTTM patients. Therapeutic anticoagulation was initiated more frequently, suggesting that this may be more practicable in these critically ill patients.

Since in our population, patients who were started with targeted therapy had significantly longer survival times, a clinically relevant difference in a disease with an otherwise rapidly fulminant trajectory becomes evident. As all patients who survived more than one week after admission were started with specific therapy, the importance of rapid initiation becomes clear. The longest overall survival time was achieved in the patient receiving chemotherapy, suggesting high efficacy of antineoplastic treatment. Most of the patients diagnosed received only therapeutic anticoagulation, which was associated with survival above the median, thus indicating a benefit of antithrombotic therapy. Our data of prolonged survival following initiation of therapy underline the importance of diagnosing PTTM and the therapeutic implications of this diagnosis. This is consistent with the literature, with emerging case reports suggesting that targeted therapeutic intervention may meaningfully prolong survival in PTTM [1, 29–31].

### Limitations

Though there is a bias caused by the small number of patients observed with different cancerous entities in a single center, to our knowledge, this is the largest consecutive monocentric series of patients with an acute presentation of PTTM. Therefore, we can assure that lab values and clinical tests were performed with the same standards. Indeed, we admitted all our patients as emergencies. None were elective deferrals, i.e., after a suspicious lung biopsy. Therefore, we observed a very accelerated course of PTTM, and its progression cannot be compared to patients with a mean life expectancy of 9.5 weeks as described in literature [1].

It is important to note that, due to the exploratory nature of our study, all results have to be interpreted as generating hypothesis rather than definitive conclusions. The small sample size limits the analyses to mainly descriptive statistics, aimed at exploring this currently largely unexplored field, and identifying potential directions for further research. Therefore, it is important to note, that larger, randomized trials are needed to validate our results.

## Conclusion

PTTM is a deadly complication of adenocarcinoma in various locations and presents with acute right heart failure in patients of every age. With low rates of obduction in Germany, the rapid onset of PTTM and the complex diagnostic process, the real incidence of PTTM remains elusive. It is often mistaken for a manifestation of CAD with sudden cardiac death as it exhibits similar risk factors, acuity and clinical presentation. The only way to improve diagnosis is to further raise awareness among medical personal in radiology, emergency, oncological, pulmonal, and cardiological departments to this emergent and lethal condition. The pattern of symptoms of right heart failure and metabolic dysregulation in the sense of obesity and diabetes should rise the suspicion. TTE as well as lung biopsy, RHC, or dual-energy CT should be performed early if available. Tumor therapy, i.e., emergency chemotherapy, antiproliferative therapy, and vasoactive therapy, is the only known effective measure yet and should be made available to PTTM patients in the right balance as they can significantly increase the survival time of this condition in case of a timely diagnosis. The role of oral antidiabetics is yet to be investigated. Especially in young patients, autopsies should be performed to identify the cancerous entity and screen relatives.

## Data Availability

All data leading to the conclusion, questioning, or supporting the generating hypothesis are included in this manuscript.
